# Development and Characterization of the PSMA-Expressing CT26-PSMA Cell Line as a Rapid Preclinical Platform for ^68^Ga-Labeled PSMA-Targeted Radioconjugates

**DOI:** 10.3390/ijms27167426

**Published:** 2026-08-19

**Authors:** Aleksandr S. Lunev, Kristina A. Petrosova, Marat G. Rakhimov, Anastasiia A. Uspenskaia, Aleksey E. Machulkin, Ipatii S. Malakhov, Olga A. Shashkova, Marina P. Samoilovich, Alexandra E. Zakharkina, Anton A. Larenkov

**Affiliations:** 1 State Research Center — Burnasyan Federal Medical Biophysical Center of Federal Medical Biological Agency, 123182 Moscow, Russia; christfmbc@gmail.com (K.A.P.); marat.rakhimov89@gmail.com (M.G.R.); alexradbio@gmail.com (A.E.Z.); 2 Faculty of Chemistry, Federal State Educational Institution of Higher Professional Education Lomonosov Moscow State University, 119991 Moscow, Russia; uspenskaya.n@gmail.com (A.A.U.); alekseymachulkin@rambler.ru (A.E.M.); 3 Department of Biochemistry, People’s Friendship University of Russia Named After Patrice Lumumba (RUDN University), 117198 Moscow, Russia; 4 A. Granov Russian Research Center for Radiology and Surgical Technologies, 197758 St. Petersburg, Russia; ipatii.malakhov@uni-luebeck.de (I.S.M.); ujinolga@yandex.ru (O.A.S.); mpsamoylovich@gmail.com (M.P.S.)

**Keywords:** PSMA, prostate cancer, CT26-PSMA, cell model, gallium-68, radiopharmaceutical, positron emission tomography, radionuclide diagnostics, preclinical study

## Abstract

Preclinical models play a critical role in the development of PSMA-targeted radiopharmaceuticals for prostate cancer. However, many existing models have practical limitations, including slow tumor growth, low engraftment rates, and restricted availability, and all human PSMA-positive lines are confined to immunodeficient hosts. We developed and characterized a novel PSMA-expressing transgenic cell line, CT26-PSMA, as a practical tool for preclinical screening of PSMA-targeting agents. The CT26-PSMA cell line was established by stable transfection of the murine colon carcinoma CT26 cell line with human PSMA using the Sleeping Beauty transposon system. PSMA expression was confirmed by RT-qPCR (reverse transcription quantitative polymerase chain reaction), flow cytometry, and radioligand saturation binding on intact cells. Two [^68^Ga]Ga-labeled radioconjugates—the well-established PSMA-617 and a newly synthesized conjugate (Conjugate-1)—were used to validate the functionality of the model through *in vitro* binding, uptake and internalization studies, and through *ex vivo* biodistribution in CT26-PSMA tumor-bearing athymic male nu/nu mice. The CT26-PSMA cell line demonstrated high and stable PSMA expression, with approximately 95% of cells expressing the biomarker and no measurable loss over 16 passages in antibiotic-free medium. Saturation binding gave a receptor density of ∼3.5 × 10^6^ sites per cell, approximately four-fold higher than that of LNCaP cells (∼0.8 × 10^6^), with dissociation constants that were indistinguishable between the two radioconjugates and between the two cell lines (K_d_ 9.0–11.6 nM). Subcutaneous tumors reached ~300 mm^3^ within 8–10 days of inoculation, with a take rate of 10/10 versus 1/10 for LNCaP (Fisher’s exact test, *p* = 1.2 × 10^−4^). Both radiotracers showed saturable, 2-PMPA-blockable binding and uptake in CT26-PSMA cells, confirming the functional activity of the recombinant receptor. Biodistribution studies revealed accumulation of both conjugates in CT26-PSMA tumors, with generally comparable tumor-to-background profiles. The CT26-PSMA cell line represents a robust, rapid, and reproducible platform for preclinical evaluation of PSMA-targeting radiopharmaceuticals, and its murine BALB/c origin permits engraftment in immunocompetent or minimally immunosuppressed hosts, whereas existing human PSMA-positive lines do not. It is intended as a screening platform rather than as a model of prostate cancer biology. The validation data obtained with [^68^Ga]Ga-labelled conjugates confirm the suitability of this cell line for future studies of PSMA-directed compounds.

## 1. Introduction

Prostate cancer (PCa) is the second most frequently diagnosed malignant neoplasm in men (14.6%) worldwide and the second leading cause of cancer death in men in most countries of the world [1]. In total, 1.55 million cases of PCa were first detected worldwide in 2024. According to Globocan, 420,000 PC-related deaths were registered worldwide in 2024. High incidence rates are traditionally noted in highly developed countries of the world (USA, Canada, Western Europe, Australia) [2,3]. It is also alarming that in recent decades, there has been a trend towards an increase in the incidence of cancer in men of relatively young and mature age, which in turn leads to an increase in mortality in this group of the population [4]. There is a wide variety of biologically active compounds that can be modified for use in targeted delivery to malignant tumors. The target for such delivery is typically a protein marker specific to a particular type of cancer [5].

The success of PCa treatment, like any disease, depends on the timeliness and completeness of its diagnosis. PCa is characterized by a number of biochemical and cellular markers that allow for identifying and monitoring the progression degree (or regression in successful therapy cases) of this disease. The presence of cellular biomarkers also allows the use of receptor-specific therapy. Biomarkers can be detected by various methods, including radionuclide methods that are suitable for the earliest and most accurate visualization of pathological processes. At least competitive non-invasive methods rely on morphophysiological changes in tissues, whereas emission tomography reflects the functional status of pathology [6].

The development of targeted radionuclide diagnostics and therapy with radiopharmaceuticals based on radiolabeled prostate-specific membrane antigen (PSMA) ligands has been a genuine breakthrough, made possible by advances in the synthetic chemistry of small-molecule targeting vectors [7]. PSMA, or glutamate carboxypeptidase II, N-acetyl-α-linked acidic dipeptidase I, or folate hydrolase, is a type II transmembrane glycoprotein belonging to the M28 family of peptidases. This protein acts as a glutamate carboxypeptidase on a variety of substrates, including nutritional folate and the neuropeptide N-acetyl-L-aspartyl-L-glutamate [8]. PSMA is one of the most representative markers of PCa because this antigen is highly and specifically expressed on the tumor surface cells developing from the prostate gland and in metastatic and hormone-refractory carcinomas at almost all stages of the disease [9]. PSMA is normally expressed in secretory cells of prostate epithelium and at substantially lower levels in several normal tissues, including the kidneys, salivary glands, and small intestine, whereas its expression in benign prostate neoplasms is negligible. Importantly, the marked quantitative difference in PSMA expression between malignant cells (high density) and normal tissues (low-to-moderate density) provides the basis for high-contrast molecular imaging using PSMA-targeted radiopharmaceuticals. It is located in the prostate cells’ cytosol and is associated with membrane proteins in malignant processes. The PSMA expression level correlates with the decreasing degree of tumor differentiation and is more often elevated in metastatic and hormone-refractory prostate tumors. Unlike prostate-specific antigen (PSA), PSMA is undetectable in blood, which makes it an almost ideal marker of prostate cancer cells and an excellent target for radionuclide imaging and therapy [10,11,12].

Preclinical studies of radiopharmaceuticals allow proving the efficacy of radiolabeled PSMA conjugates at the development stage, but for this purpose, it is necessary to select a relevant model for *in vitro* and *in vivo* studies. Different cell types are used as preclinical PCa models, each with their own distinct features. A range of preclinical prostate cancer models is available, and the choice of cell line in radiopharmaceutical research is determined principally by the molecular target rather than by the tumor type: PC3 and DU-145 are the standard models for GRPR-targeted agents, whereas PSMA-directed studies rely predominantly on the androgen-dependent LNCaP line and on PSMA-transfected derivatives such as PC3-PIP [13].

LNCaP is a cell line derived from lymph node metastasis. The LNCaP xenograft model, widely used to study prostate cancer, involves implanting LNCaP tumor cells into immunodeficient mice, such as nu/nu or NOD/SCID strains. This model has been instrumental in evaluating the biology of PCa and the efficacy of various treatments. It harbors a T877A androgen-resistant mutation, with ligand promiscuity, and has only a modest potential to form xenografts in nude mice [14]. It has multiple derivative cell lines, differing from the maternal LNCaP cell lines in different ways: C4-2b (PSA +, bone metastatic in mice), LNCaP-abl (androgen-resistant, androgen-depleted medium), LNCaP-AR (androgen-resistant, tissue culture), ResA and B (androgen- and anti-androgen-resistant) [15]. VCaP cells come from a metastatic lesion and have a wildtype, yet amplified, AR. VCaP cells are especially interesting in preclinical research as they express the TMPRSS2-ERG gene arrangement, commonly seen in PCa patients [16]. DuCaP is derived from a hormone-refractory xenograft of a dura mater metastasis and expresses PSA and overexpresses wildtype AR [17]. Multiple LAPC cell lines are androgen-responsive and developed from a xenograft of a PCa lymph node (LAPC-4) and bone (LAPC-9) metastasis. LAPC-4 is characterized by mutations of p53 and wildtype PTEN [18]. DU-145 is derived from a PCa brain metastasis. It does not express AR and is therefore hormone-insensitive. PC3 cells are grown from a PCa bone metastasis and are, similarly to DU-145, AR-negative [19]. Some studies suggested that PC3 cells have neuroendocrine features reflecting small-cell PCa and stem-like features [20]. 22Rv1 is a cell line derived from a relapsing xenograft of the CWR22 cell line in mice that were castrated [21]. It harbors the AR H874Y mutation and, due to an intragenic duplication of exon 3, also expresses a truncated AR isoform; its PTEN status is wildtype. It is mostly used in research involving splice variants of the AR [22]. MDA PCa 2A is a cell line derived from bone metastasis, being AR-positive, harboring AR mutants L702H and T878A [23].

However, while LNCaP xenografts offer valuable insights, challenges arise due to their relatively low take rate (success of tumor establishment) and variability in tumor growth. These factors make the model less efficient for high-throughput drug screening and can lead to inconsistent results in translational research [24,25,26,27]. In response to these limitations, researchers have increasingly turned to alternative tumor cell lines or more advanced models, such as the DU145 or PC3 lines, which are more aggressive and have higher engraftment success rates. Additionally, patient-derived xenograft (PDX) models have become popular for their ability to closely mimic human tumors, including genetic and histological fidelity across generations. PDX models are also paired with humanized mouse models, allowing researchers to study human tumor interactions with the immune system, further improving the relevance of preclinical studies in cancer drug development [28].

In recent years, PSMA-expressing transgenic cell lines have emerged as valuable alternatives to traditional xenograft models for prostate cancer research. By engineering PSMA-expressing cells, researchers can create more relevant and consistent models to test the efficacy of PSMA-targeted therapies. One notable example is the PC3-PIP cells, which represent a modified version of the PC3 prostate cancer cell line that stably expresses PSMA [29]. While PC3 cells, derived from a bone metastasis of a grade IV prostate cancer, naturally lack PSMA expression, PC3-PIP cells have been engineered to express this antigen, making them a more relevant model for studying PSMA-targeted therapies. The expression of PSMA in PC3-PIP cells allows researchers to investigate the efficacy of PSMA-targeted radioligand therapies, antibodies, and small molecules, which would not be as feasible in native PC3 cells. This makes the PC3-PIP cell line a valuable tool for preclinical research in prostate cancer treatment, especially for therapeutic approaches involving PSMA-specific targeting. Two practical limitations of the PC3-PIP model motivated the present work. First, it is not commercially available and is distributed through material transfer agreements between individual laboratories, so access depends on personal contact and on institutional agreements that can take considerable time to conclude. Second, and more fundamentally, PC3-PIP is a human cell line and can therefore be propagated only in athymic or otherwise immunodeficient recipients. The same restriction applies to LNCaP, 22Rv1, VCaP, and C4-2. As a consequence, no established PSMA-positive tumor model permits the study of PSMA-targeted agents in a host with an intact immune system, and questions that require one—a combination of radioligand therapy with immunotherapy, PSMA-directed cellular therapies, or radiation-induced immunogenic cell death—cannot at present be addressed in a PSMA-expressing tumor.

The CT26 cell line is a murine colon carcinoma cell line widely used in cancer and immunology research. These cells originate from BALB/c mice and are known for their high immunogenicity, making them ideal for modeling tumor growth, studying immune response mechanisms, and testing anticancer therapies in preclinical models. The CT26 line is used to create various tumor models in mice, enabling researchers to explore tumor–immune system interactions, the efficacy of chemotherapy drugs, and immunotherapy methods [30].

Transgenic CT26-PSMA cells expressing human PSMA were engineered to develop and test PSMA-targeted pharmaceuticals intended for visualization and treatment of PC through specific immunotherapeutic approaches, such as CAR-T cells, conjugates of antibodies, or small molecules with radionuclides or cytotoxins.

The use of PSMA-expressing transgenic models is particularly beneficial in preclinical testing of PSMA-targeted drugs, as they allow for precise validation of drug effects on prostate cancer cells with known receptor expression levels. This leads to more reliable predictions of clinical efficacy, further supporting the trend toward more specialized and molecularly defined cancer models. The development of more robust cell models is part of a broader strategy to improve the reliability of preclinical studies, especially when traditional lines like LNCaP exhibit unpredictable behavior. So, creating and using new stable transgenic PSMA-expressed cells and rapidly forming tumors for preclinical studies of new radiolabeled PSMA conjugates is a necessary and promising task.

## 2. Results

### 2.1. Radiolabeling

The radioconjugates [^68^Ga]Ga-PSMA-617 and [^68^Ga]Ga-Conjugate-1 were obtained with radiochemical purity of ≥96% in the crude, sterile-filtered reaction mixture, i.e., in the preparation as administered, with no post-labeling purification having been performed (for corresponding radio–TLC and radio–HPLC chromatograms, see Supplementary Materials Figures S2–S4) and molar activity of 39–198 MBq/nmol and 54–273 MBq/nmol, respectively.

### 2.2. PSMA-Expressing Cells Characterization

The expression level of the human PSMA gene in the transgenic cells 55 days after transfection corresponded to ΔCt = −0.53, i.e., close to that of the housekeeping gene GAPDH, and did not change measurably over the following 16 passages in medium free of selective antibiotic. The corresponding value for LNCaP cells was ΔCt = 2.45, so that PSMA transcript abundance in CT26-PSMA cells is approximately 7.9-fold higher than in LNCaP cells (ΔCt = 2.98).

**Figure 1:**
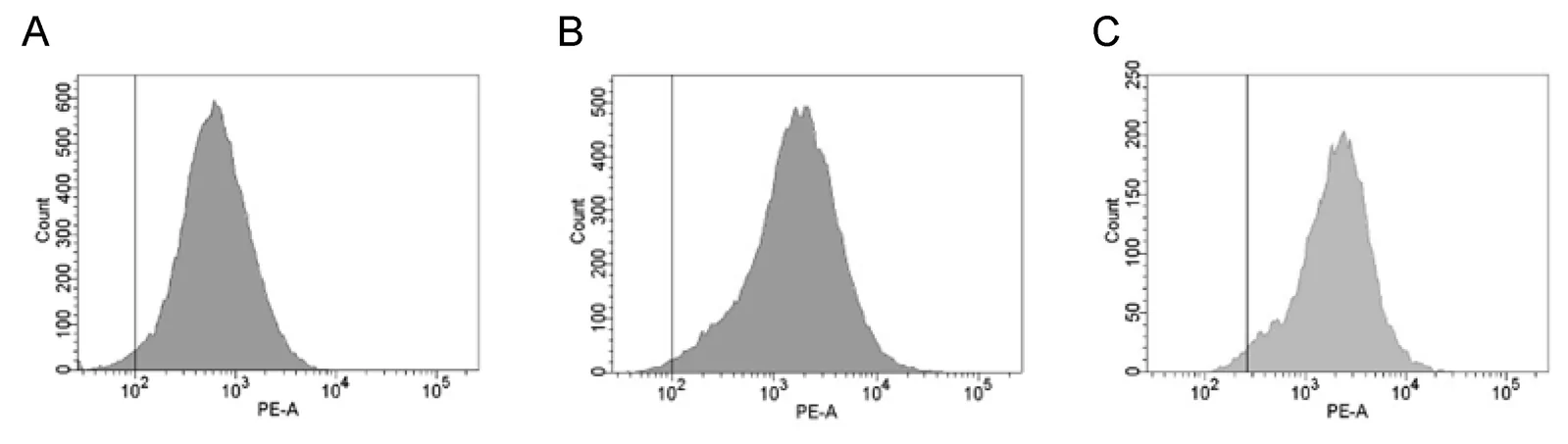
Comparison of PSMA expression on LNCaP cells (**A**) and transgenic CT26-PSMA cells; (**B**) 16 passages of cultivation in puromycin-containing culture medium and (**C**) 16 passages of cultivation in puromycin-free culture medium. Horizontal axis—conventional units of fluorescence intensity; vertical axis is the number of cells. The level of isotypic control is indicated by a vertical line. Data were obtained with anti-PSMA antibody clone BL 342504 (BioLegend, USA); the level of isotype control is indicated by a vertical line.

The number of cells expressing human PSMA and its density on cell membranes were evaluated by flow cytofluorimetry. A higher proportion of CT26-PSMA cells carried the biomarker than human prostate adenocarcinoma cells of the LNCaP line (95% for CT26-PSMA, 86% for LNCaP). The fluorescence signal intensity of CT26-PSMA cells was 2.6 times higher than the values obtained for human cells, indicating a higher density of PSMA molecules on the cell membrane of the transgenic line (Figure 1).

### 2.3. In Vitro Binding Characteristics and Specificity of the PSMA-Targeted Radioconjugates

**Figure 2:**
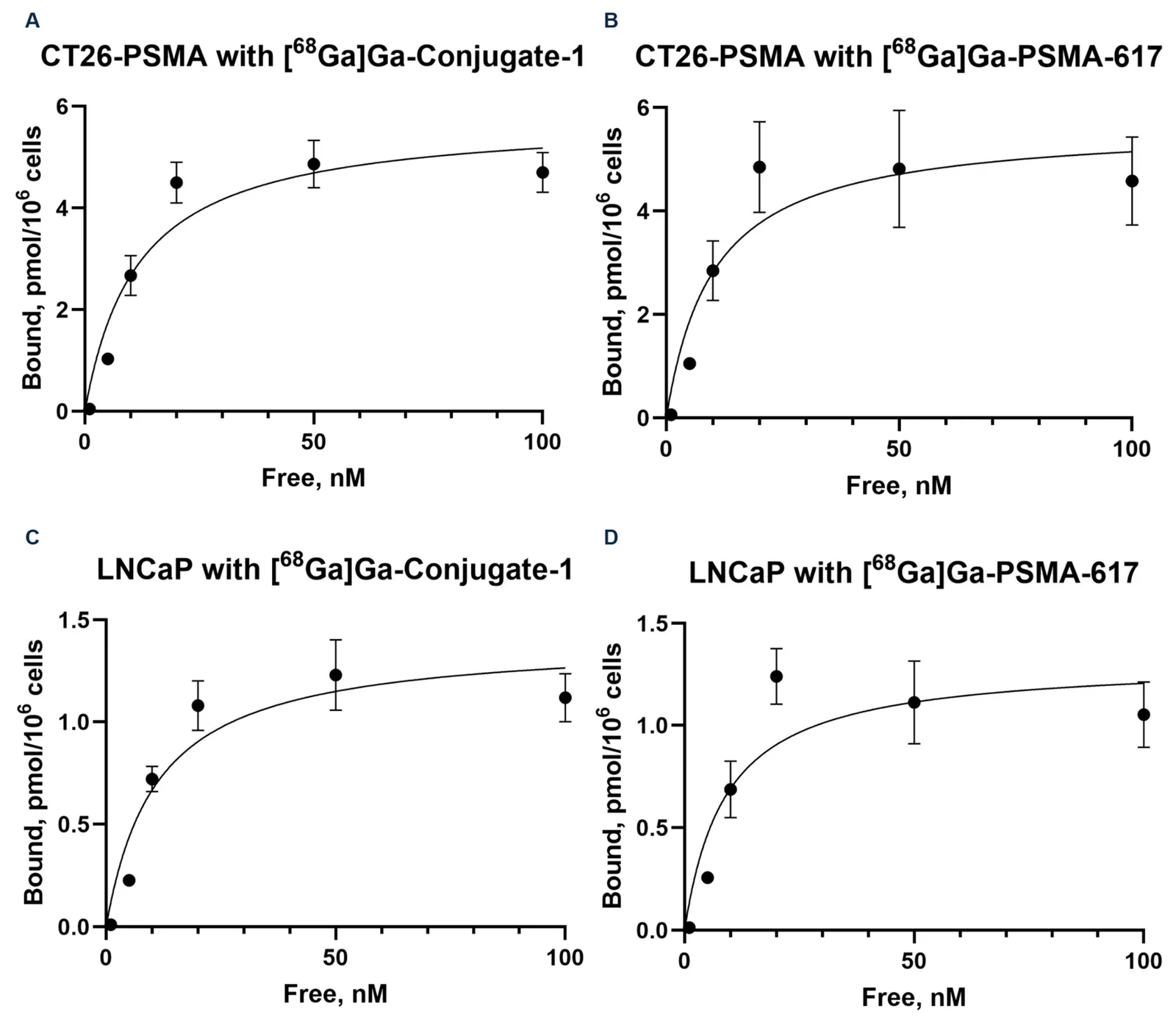
Saturation binding curves: CT26-PSMA cells with [^68^Ga]Ga-Conjugate-1 (**A**), CT26-PSMA cells with [^68^Ga]Ga-PSMA-617 (**B**), LNCaP cells with [^68^Ga]Ga-Conjugate-1 (**C**), and LNCaP cells with [^68^Ga]Ga-PSMA-617 (**D**). Curves represent specific binding (total minus non-specific, with the latter determined in the presence of 1 µM 2-PMPA); three technical replicates in each of two independent experiments.

The cytofluorimetric data indicate that CT26-PSMA cells carry the biomarker on a larger proportion of cells and at a higher membrane density than the widely used LNCaP line, which makes them a practical alternative model for studies involving PSMA. To determine the receptor density on the surface of CT26-PSMA and LNCaP cells, saturation binding curves were generated, and the K_d_ and B_max_ values were calculated (Figure 2).

Saturation binding data are presented in Table 1.

To quantify PSMA expression levels and compare the binding characteristics of the two radiotracers, saturation binding assays were performed on CT26-PSMA and LNCaP cells. The calculated K_d_ values for [^68^Ga]Ga-Conjugate-1 and [^68^Ga]Ga-PSMA-617 were 11.6 nM and 10.1 nM, respectively, on CT26-PSMA cells, and 9.1 nM and 9.0 nM, respectively, on LNCaP cells, indicating comparable binding affinities between the two conjugates on each cell line. Notably, the B_max_ values for CT26-PSMA cells were approximately four-fold higher (5.8 (95% CI 5.0–6.7) and 5.7 (4.6–7.0) pmol/10^6^ cells for Conjugate-1 and PSMA-617, respectively) than those for LNCaP cells (1.4 (1.2–1.7) and 1.3 (1.1–1.6) pmol/10^6^ cells), corresponding to an average receptor density of approximately 3.5 × 10^6^ receptors per cell for CT26-PSMA versus 0.8 × 10^6^ receptors per cell for LNCaP (a 4.1-fold difference with Conjugate-1 and a 4.4-fold difference with PSMA-617). These findings are concordant in direction with the flow cytometry data; the difference in magnitude (2.6-fold by mean fluorescence intensity versus approximately four-fold by B_max_) is expected, since antibody-derived fluorescence intensity is not a linear function of the receptor number at high receptor densities. Furthermore, the nearly identical K_d_ values obtained for both conjugates on each cell line suggest that Conjugate-1 and PSMA-617 recognize the same binding sites on the PSMA receptor, which is consistent with the proper conformational integrity of the recombinant receptor on the surface of the CT26-PSMA cells. Importantly, the calculated receptor density for the reference LNCaP cell line (~0.8 × 10^6^ receptors/cell) falls within the published range (0.5–1.4 × 10^6^), which serves as a strong external validation of our saturation binding methodology [31].

**Figure 3:**
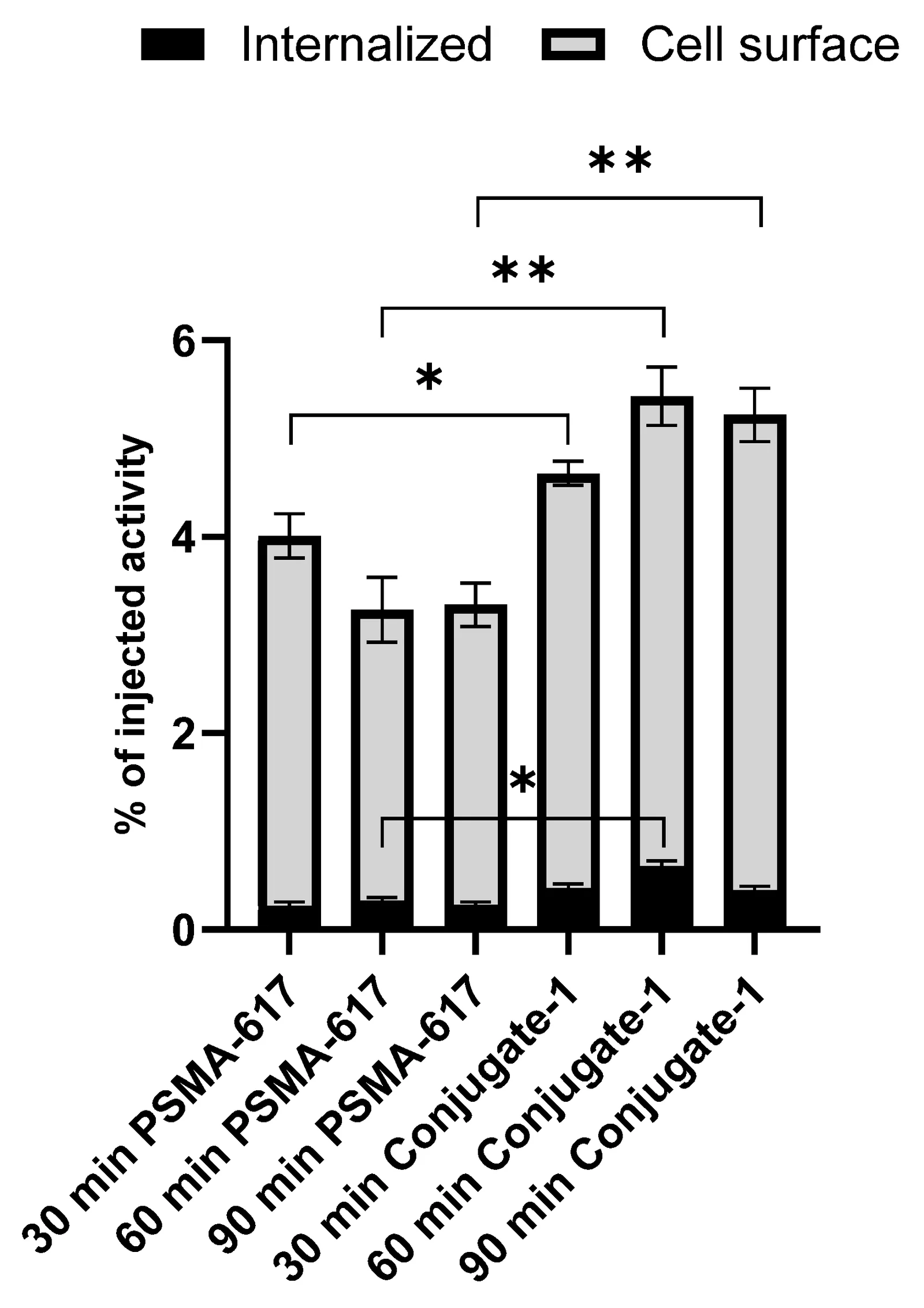
Histogram of *in vitro* uptake (mean ± SEM, n = 5) of [^68^Ga]Ga-labeled conjugates in CT26-PSMA cell culture. * (*p* < 0.05) and ** (*p* < 0.01) represent a significant difference between [^68^Ga]Ga-PSMA-617 and [^68^Ga]Ga-Conjugate-1 uptake. Comparisons were made with the exact Mann–Whitney U-test; with n = 5 per group, the smallest attainable two-sided *p*-value is 0.0079, so *p* < 0.01 is the highest attainable significance level.

The novel human PSMA-expressing CT26-PSMA cell line had been used for comparative evaluation of the accumulation and internalization of [^68^Ga]Ga-labeled conjugates PSMA-617 and Conjugate-1 (Figure 3).

The data from total uptake and internalization experiments have been included as Supplementary Table S1. Cell-associated activity was higher for [^68^Ga]Ga-Conjugate-1 than for [^68^Ga]Ga-PSMA-617 at all three time points, whereas the internalized fractions, expressed as a percentage of total cell-associated activity, were comparable between the two radioconjugates. Both radioconjugates therefore bind and are internalized by the recombinant receptor, which is the property required of the model. The modest absolute internalized fractions observed for both tracers over the 90 min window most likely reflect a dynamic equilibrium between internalization, receptor recycling, and degradation of the ligand–receptor complex, and, given the 67.71 min physical half-life of ^68^Ga, may represent only the early phase of a slower process rather than a lack of specific binding. These findings support the utility of the CT26-PSMA model for preclinical screening and provide a rationale for future *in vivo* studies to assess the potential of Conjugate-1 in prostate cancer and other PSMA-expressing malignancies.

### 2.4. In Vivo Studies

**Figure 4:**
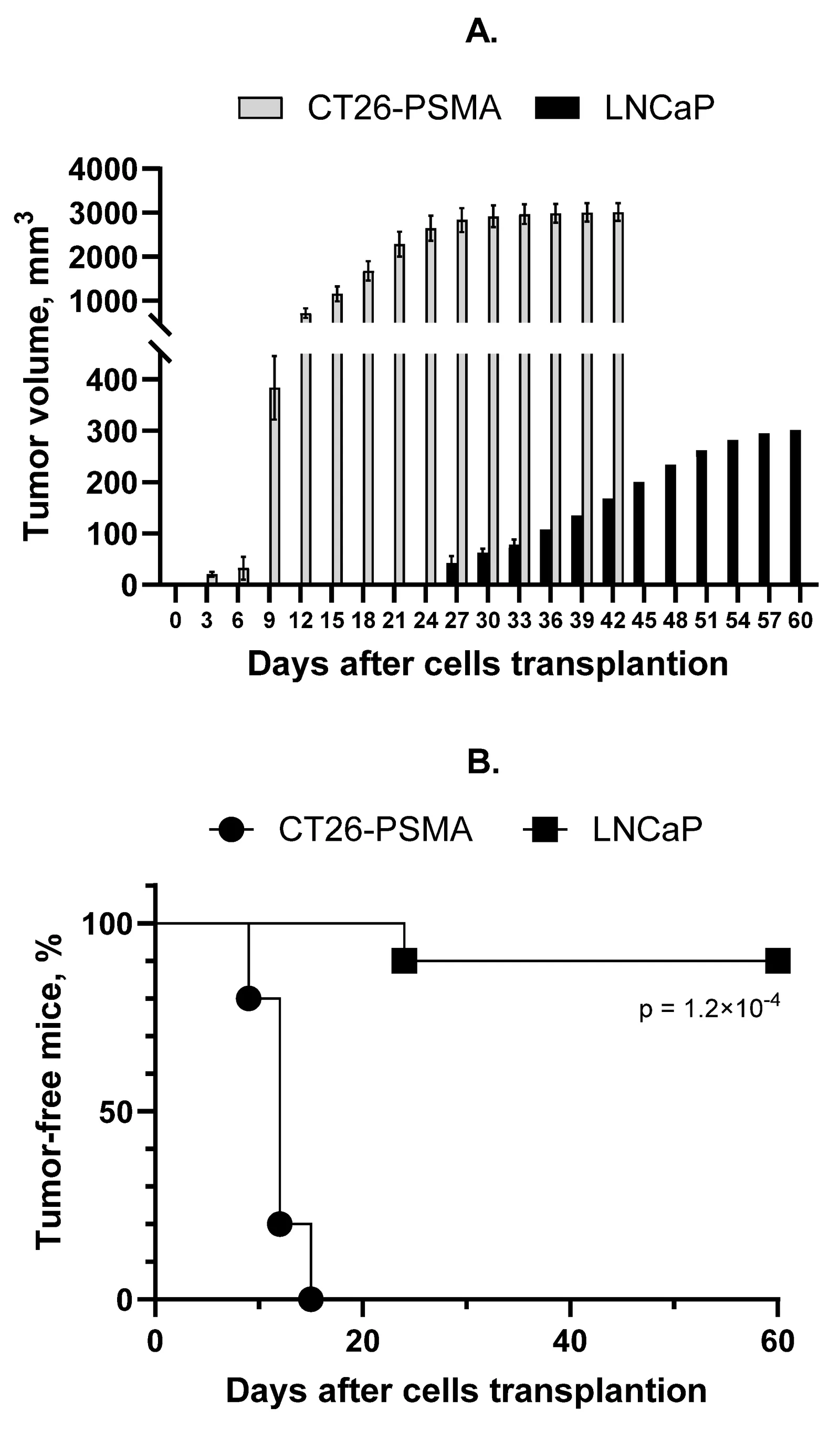
Growth kinetics of subcutaneous LNCaP and CT26-PSMA tumors in immunodeficient mice (**A**) and tumor engraftment kinetics of LNCaP and CT26-PSMA cells in mice (**B**) (mean ± SEM, n = 10).

Subcutaneous CT26-PSMA tumors (~300 mm^3^) had been forming for 8–10 days after inoculation of CT26-PSMA cells into the mice. The survival rate and tumor formation are close to 100%. The CT26-PSMA model offers rapid and reproducible tumor formation in immunodeficient mice, in contrast to the considerably slower growth kinetics of LNCaP xenografts (Figure 4).

Two cell lines, LNCaP and CT26-PSMA, were subcutaneously implanted into athymic nu/nu mice (n = 10 per group). In the LNCaP group, successful tumor engraftment and xenograft development occurred in only one mouse (10% take rate). Moreover, the growth was exceptionally slow: a palpable tumor appeared only 27 days post-inoculation, and by day 60, the tumor volume had reached just 300 mm^3^. In contrast, palpable CT26-PSMA tumors emerged in some mice as early as 8–10 days after implantation, and by day 15, all ten mice had developed palpable tumors, yielding a 100% engraftment rate (histology of CT26-PSMA tumor is presented in Supplementary Materials Figure S5). In contrast, in the LNCaP group, successful tumor engraftment occurred in only one mouse (10% take rate; Fisher’s exact test, two-sided *p* = 1.2 × 10^−4^ for 10/10 versus 1/10). The CT26-PSMA tumors displayed aggressive and rapid growth, exceeding 2000 mm^3^ within approximately three weeks. Such large tumor volumes are considered unacceptable for most *in vivo* studies, as they are frequently accompanied by necrotic foci, lead to heterogeneous accumulation of radiolabeled ligands or therapeutics, and raise significant animal welfare concerns, requiring early euthanasia. Thus, while the CT26-PSMA line offers the advantages of rapid tumor growth and complete engraftment compared to LNCaP, its extremely fast progression imposes a narrow window and strict time limitations for experimental use. This should be taken into account when planning experiments.

**Figure 5:**
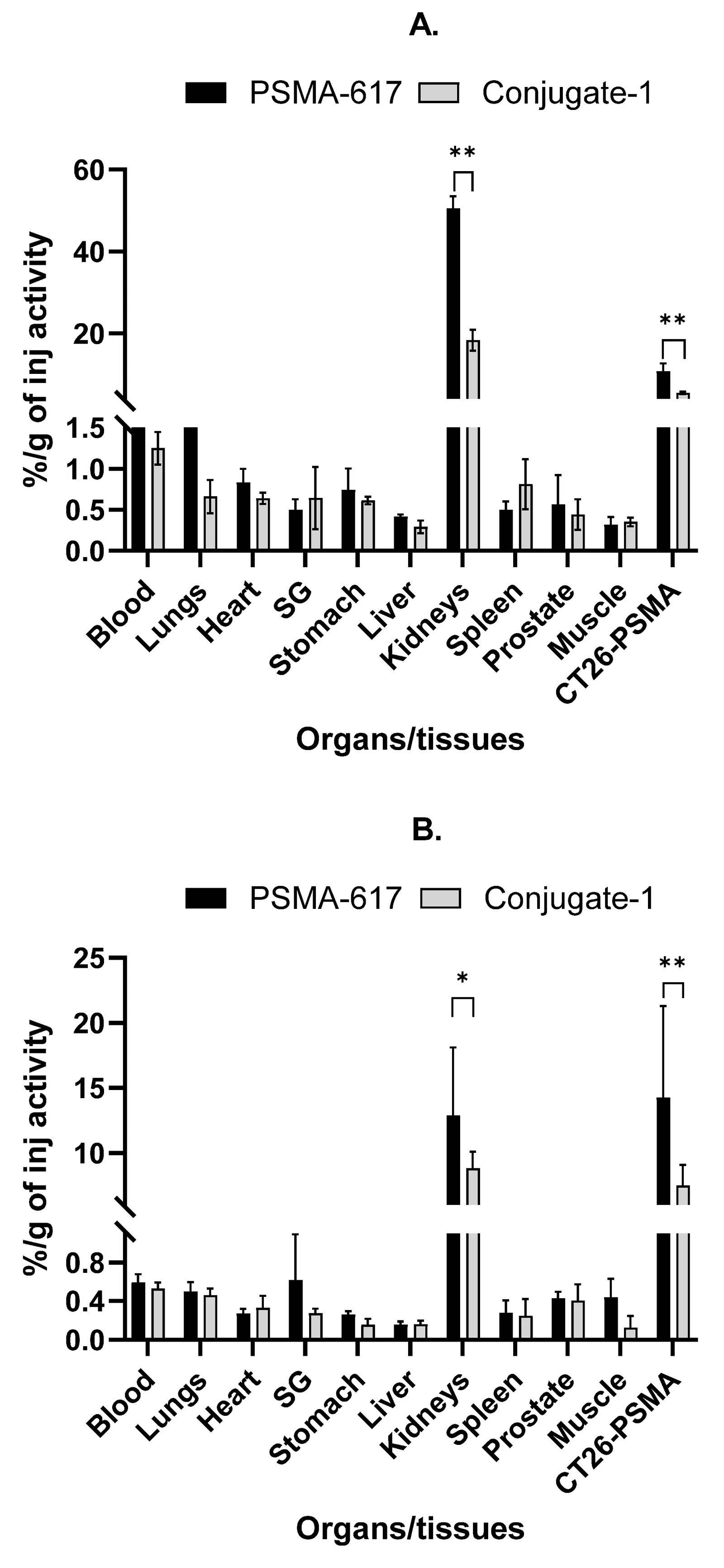

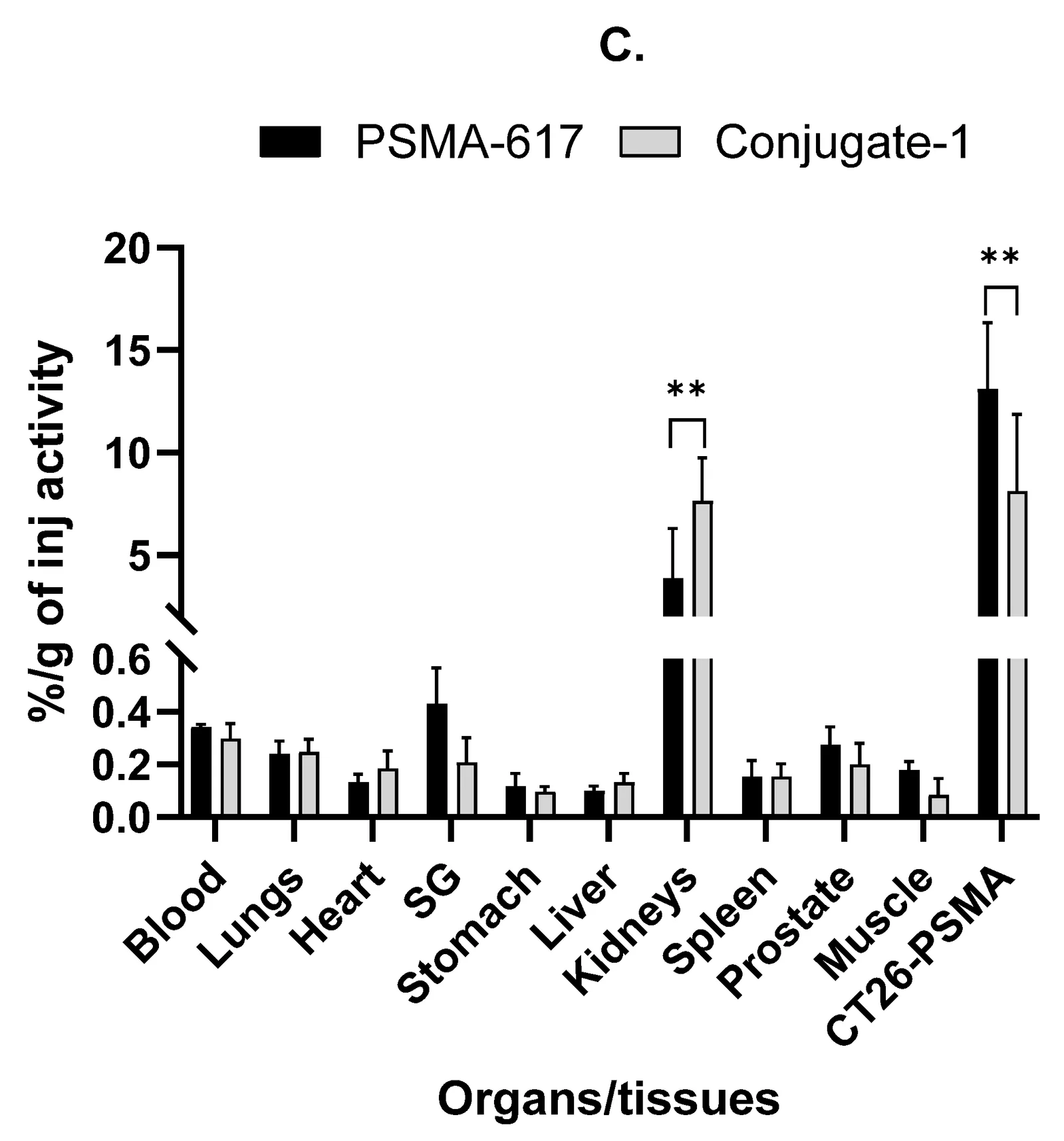
Histogram of *in vivo* uptake (% IA/g) of [^68^Ga]Ga-labeled conjugates in athymic mice (mean ± SEM, n = 5): 30 min after i.v. injection (**A**), 60 min after i.v. injection (**B**), and 90 min after i.v. injection (**C**). * (*p* < 0.05) and ** (*p* < 0.01) represent a significant difference between [^68^Ga]Ga-PSMA-617 and [^68^Ga]Ga-Conjugate-1 uptake in the organs indicated. SGs—salivary glands. Comparisons were made with the exact Mann–Whitney U-test; with n = 5 per group, the smallest attainable two-sided *p*-value is 0.0079, so *p* < 0.01 is the highest attainable significance level.

Numerical values of *in vivo* uptake of [^68^Ga]Ga-labeled conjugates (PSMA-617 and Conjugate-1) in athymic mice have been included as Supplementary Table S2. At 30 min after intravenous injection, the activity concentration in the kidneys and in the CT26-PSMA tumor exceeded that in blood for both radioconjugates, which is favorable for tumor-to-background contrast on PET imaging. There was a statistically significant difference between tumor uptake throughout the study for [^68^Ga]Ga-labeled conjugates (PSMA-617 and Conjugate-1). The time of 60–90 min is the most appropriate for PET imaging of pathology foci (Figure 5).

Based on the biodistribution data presented in Figure 5, [^68^Ga]Ga-Conjugate-1 demonstrated significantly lower uptake in the kidneys compared to [^68^Ga]Ga-PSMA-617 at 30 and 60 min post-injection, although this difference diminished and showed a slight reversal at 90 min. However, when considering the cumulative area under the curve (AUC) for renal uptake, the overall radiation exposure to the kidneys was still considerably lower for [^68^Ga]Ga-Conjugate-1 compared to [^68^Ga]Ga-PSMA-617, which may translate into a more favorable renal dosimetry, although no dosimetric calculation was performed in the present study.

Regarding tumor accumulation, [^68^Ga]Ga-Conjugate-1 consistently exhibited lower absolute uptake values than [^68^Ga]Ga-PSMA-617 across all time points. Despite this lower absolute tumor uptake, the tumor-to-blood and tumor-to-muscle ratios for [^68^Ga]Ga-Conjugate-1 were comparable to or slightly higher than those of [^68^Ga]Ga-PSMA-617, due to the concomitantly reduced background activity.

Direct evidence of diagnostic performance can be obtained only from preclinical PET/CT imaging, which was not performed in this study. In the absence of such data, we therefore regard [^68^Ga]Ga-Conjugate-1 as a candidate warranting further evaluation and draw no conclusion regarding its imaging performance relative to [^68^Ga]Ga-PSMA-617.

**Figure 6:**
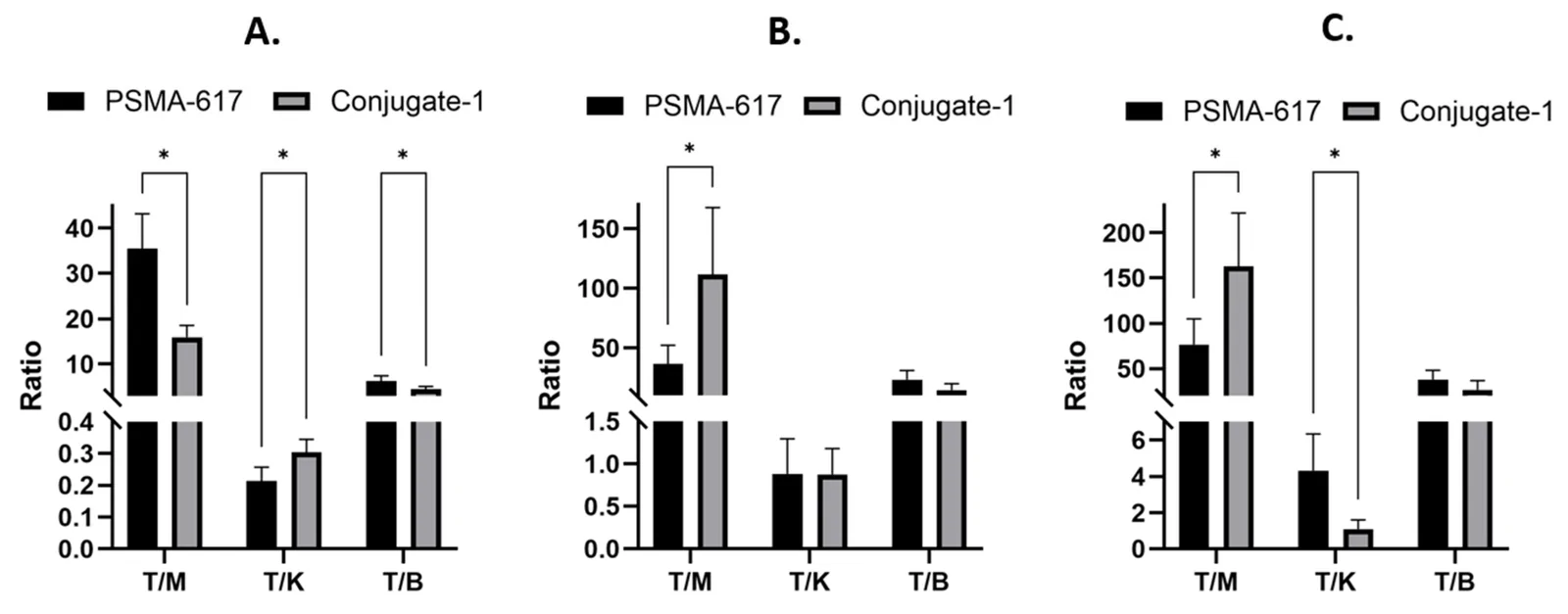
Ratio data of [^68^Ga]Ga-labeled conjugates in athymic mice (mean ± SEM, n = 5): 30 min after i.v. injection (**A**), 60 min after i.v. injection (**B**), and 90 min after i.v. injection (**C**). * (*p* < 0.05) represents a significant difference between [^68^Ga]Ga-PSMA-617 and [^68^Ga]Ga-Conjugate-1 ratios. T/M—tumor-to-muscle ratio, T/K—tumor-to-kidney ratio, T/B—tumor-to-blood ratio. Comparisons were made with the exact Mann–Whitney U-test; with n = 5 per group, the smallest attainable two-sided *p*-value is 0.0079, so *p* < 0.01 is the highest attainable significance level.

To assess the imaging potential of both radiotracers, the tumor-to-muscle, tumor-to-kidney, and tumor-to-blood ratios were calculated based on the biodistribution data (Figure 6).

At 30 min post-injection, the tumor-to-muscle ratio was significantly higher for PSMA-617 (35.5 ± 7.7 vs. 15.9 ± 2.6; *p* < 0.05). However, at 60 and 90 min, Conjugate-1 exhibited significantly higher tumor-to-muscle ratios compared to PSMA-617 (60 min: 111.5 ± 56.3 vs. 36.4 ± 15.8, *p* < 0.05; 90 min: 142.9 ± 87.0 vs. 76.6 ± 28.4, *p* < 0.05). These later values should be interpreted with caution: the muscle activity concentration in the Conjugate-1 group was 0.13 ± 0.12 and 0.08 ± 0.06% IA/g at 60 and 90 min, so the ratio is formed on a small and highly variable denominator. For tumor-to-kidney and tumor-to-blood ratios, significant differences were observed at 30 and 90 min and at 30 min, respectively. These data suggest that both tracers provide adequate tumor-to-background contrast, with Conjugate-1 demonstrating comparable performance to PSMA-617 in the context of the CT26-PSMA model.

**Figure 7:**
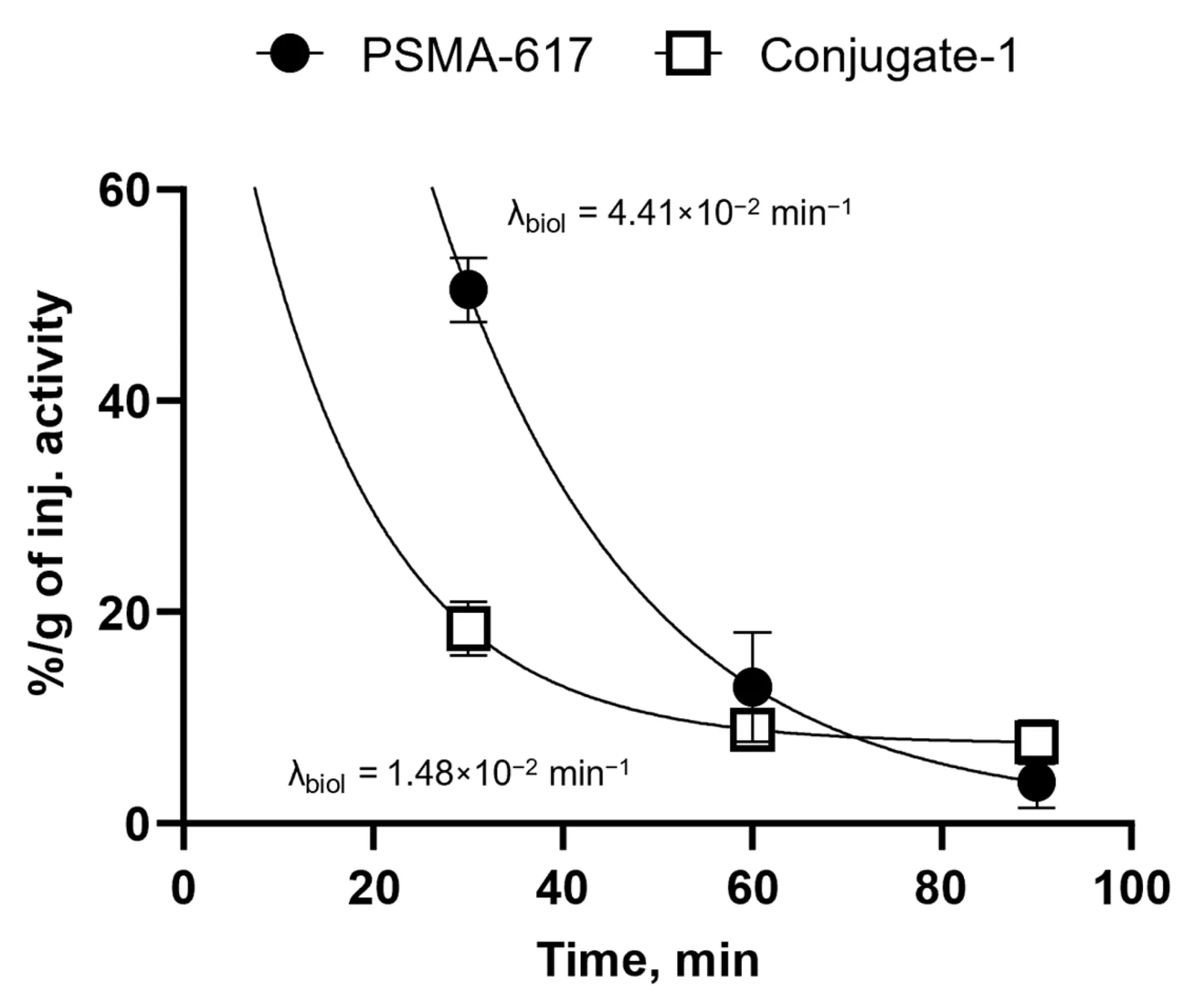
Linearization of kidney excretion for [^68^Ga]Ga-labeled conjugates in athymic mice (mean ± SEM, n = 5).

[^68^Ga]Ga-PSMA-617 kidney uptake in 30 min after i.v. injection (50.47 ± 3.06% IA/g) is much more than [^68^Ga]Ga-Conjugate-1 uptake (18.41 ± 2.55% IA/g) but the effective half-time of [^68^Ga]Ga-PSMA-617 in the kidneys is approximately two times shorter than that of [^68^Ga]Ga-Conjugate-1: 12.9 ± 1.3 and 27.5 ± 1.3 min, respectively (Figure 7).

It seemed that [^68^Ga]Ga-Conjugate-1 would generate a higher radiation dose on the kidneys than [^68^Ga]Ga-PSMA-617 because of its slower excretion, but the value of absorbed doses depends not so much on biological half-time as on the total number of organ/tissue radionuclide decays, whose value depends on the clearance of the radiolabeled conjugate (Table 2).

For the purpose of pharmacokinetic calculations, tissue density was assumed to be 1 g/mL for both tumor and kidney samples. Pharmacokinetic analysis revealed that, despite comparable blood effective half-times (18.7–20.3 min), [^68^Ga]Ga-Conjugate-1 showed significantly higher blood clearance than [^68^Ga]Ga-PSMA-617 (*p* < 0.01) and a lower blood AUC (*p* < 0.01). In the kidneys, Conjugate-1 exhibited a significantly longer effective half-time (27.5 vs. 12.9 min; *p* < 0.01) but a 3.5-fold lower AUC (*p* < 0.01) and, consequently, a higher apparent clearance (*p* < 0.01). These two observations are not contradictory: the effective half-time describes the rate at which activity is eliminated once it has accumulated, whereas the AUC—and hence the clearance derived from it—is dominated by the much lower peak renal uptake of Conjugate-1 (18.41 vs. 50.47% IA/g at 30 min). The integrated renal exposure is therefore lower for Conjugate-1 despite its slower renal elimination. In the tumor, Conjugate-1 demonstrated faster clearance and a significantly lower AUC compared to PSMA-617 (*p* < 0.01 for both). It should be noted that within the 90 min observation period, it was not possible to reliably calculate the biological half-lives of either conjugate from tumor tissue, as the clearance phase had not yet been established within this timeframe. Therefore, for the purposes of this study, it is reasonable to assume that the effective half-lives in the tumor approximate the physical half-life of gallium-68 (T_phys_ = 67.71 min).

## 3. Discussion

The results of this study provide valuable insights into the expression and targeting of the human PSMA gene in transgenic cells, as well as the comparative efficacy of two radiolabeled conjugates ([^68^Ga]Ga-PSMA-617 and [^68^Ga]Ga-Conjugate-1), in both *in vitro* and *in vivo* settings. The findings have significant implications for the development of targeted imaging and therapeutic strategies for PSMA-expressing malignancies, particularly prostate cancer.

The transgenic CT26-PSMA cell line demonstrated substantially higher PSMA expression compared to the LNCaP cells, both in terms of the percentage of cells expressing PSMA and the density of PSMA molecules on the cell membrane. This higher expression level, coupled with the robust fluorescence signal intensity, supports the utility of the CT26-PSMA model as a reliable and efficient system for preclinical evaluation of radiolabeled PSMA conjugates.

It is important to acknowledge, however, that the CT26-PSMA model is a genetically engineered murine colon carcinoma cell line rather than a prostate cancer cell line. As such, it does not fully recapitulate the tumor microenvironment, stromal interactions, or the complex biological behavior of human prostate cancer. In this regard, the CT26-PSMA model shares the same fundamental limitation as other commonly used xenograft models, including LNCaP and PC3-PIP, which also grow in a murine stromal environment and cannot fully reproduce the heterogeneity and microenvironment of human prostate tumors. Therefore, the CT26-PSMA model should be viewed primarily as a tool for rapid preclinical screening of PSMA-targeting ligands and for studying receptor-specific interactions and internalization dynamics, rather than as a model for investigating prostate cancer biology per se. Nevertheless, for the purpose of this study—comparative evaluation of radiotracer binding and specificity—the CT26-PSMA model provides a robust, reproducible, and high-throughput platform that effectively complements the classical LNCaP-based assays.

Comparative characterization of various parameters of the most popular cell cultures used in studies of radiolabeled PSMA conjugates is presented in Table 3.

As shown in Table 3, the CT26-PSMA cell line offers several practical advantages over existing models, including the shortest doubling time (24 h), the highest xenograft take rate (nearly 100%), and the most rapid tumor formation (8–10 days to ~300 mm^3^), while maintaining high PSMA expression (95% of cells). These features make it particularly suitable for high-throughput preclinical screening of PSMA-targeted radioconjugates. Its distinguishing property, however, is not throughput but host compatibility: because CT26 is derived from BALB/c mice, CT26-PSMA can be engrafted not only in athymic recipients but also in immunocompetent or minimally immunosuppressed BALB/c-background hosts, as we have demonstrated in F1 (DBA × BALB/c) mice following low-dose whole-body irradiation [48]. All of the human lines compared in Table 3 are categorically restricted to immunodeficient recipients. This opens to PSMA-targeted radiopharmaceutical research a class of experiment—a combination of immunotherapy, PSMA-directed cellular therapy, and radiation-induced immunogenic cell death—that cannot presently be addressed in a PSMA-expressing tumor. The corresponding caveat should be stated in the same breath: human PSMA is a xenoantigen in an immunocompetent mouse, and the humoral and cellular responses it elicits may limit the duration of such studies. This capability therefore requires further characterization and is presented here as a distinguishing property of the model rather than as an established equivalence to a fully syngeneic tumor.

The two radioconjugates dissociate between *in vitro* and *in vivo* behavior in a way that is itself informative. Their dissociation constants and maximal binding capacities are indistinguishable on both cell lines, and *in vitro* cell-associated activity was higher for [^68^Ga]Ga-Conjugate-1; yet its tumor uptake *in vivo* was approximately half that of [^68^Ga]Ga-PSMA-617 at every time point. Neither lower affinity nor lower receptor occupancy can therefore account for the difference, which is more plausibly attributable to systemic delivery—blood clearance, lipophilicity, and plasma protein binding—than to target engagement. [^68^Ga]Ga-PSMA-617 also showed higher initial renal uptake, while [^68^Ga]Ga-Conjugate-1 was eliminated from the kidneys approximately 2.1 times more slowly, yet with 3.5-fold lower integrated renal exposure. A model in which such a dissociation can be identified within a single experimental series is precisely the utility we claim for the CT26-PSMA platform. A direct comparison against the established diagnostic agent [^68^Ga]Ga-PSMA-11 and salivary gland dosimetry with a therapeutic radionuclide are the natural and planned next steps.

## 4. Materials and Methods

### 4.1. Chemicals and Reagents

Only 18.2 MΩ·cm deionized water (Milli-Q Millipore, Merck, Darmstadt, Germany) was used. All chemicals and solvents used in the synthesis were of ultrahigh purity or pharmaceutical grade. Hydrochloric acid solution (30% Ultrapure; Supelco/Merck, St. Louis, MO, USA) and sodium acetate (≥99.999% trace metal basis, anhydrous; Honeywell, Seelze, Germany) were used to prepare the reaction media with the required pH. The precursor PSMA-617 was kindly provided by Jenguro Ltd. (Moscow, Russia). All organic solvents used for TLC/HPLC analysis were of HPLC gradient grade.

### 4.2. Conjugate-1 Synthesis

In addition to the PSMA-617 taken for biological studies, we synthesized a new urea-based conjugate with a para-carboxy aromatic substituent at the ε-nitrogen atom of lysine and a dipeptide linker L-Tyr-L-Tyr—Conjugate-1 (the structural formula is given in Supplementary Figure S1) [49]. The detailed description of the synthesis, as well as NMR, LC-MS, and ESI-HRMS results, can be found in our work on a series of related conjugates, including Conjugate-1 [49]. In brief, the conjugate was prepared by peptide solid-phase synthesis on 2-CTC resin with 1,3-diaminopropane residue previously immobilized on it. This strategy allows for obtaining a conjugation-ready ligand that contains a free amino group in the linker structure. Conjugation was performed by classical peptide synthesis in solution, followed by removal of tert-butyl protecting groups from the urea and DOTA chelator vector fragments [50].

### 4.3. Radiochemical Synthesis and QC

A ^68^Ge/^68^Ga generator (Cyclotron Co., Ltd., Obninsk, Russia) with an initial activity of 1850 MBq was used. Post-processing of generator eluate was performed with the HCl–ethanol method [51]. The final purified and concentrated solution of ^68^Ga was obtained in 0.1 M HCl_aq._ medium. Synthesis of ^68^Ga radioconjugates was performed according to the standard procedure. In brief, to a 2.0 mL Eppendorf test tube containing an aliquot (5–20 μL) of an aqueous ligand solution (1 mg/mL), an aqueous solution of sodium acetate (2.0 M, 150–200 μL), and conditioned (purified and concentrated) eluate of the ^68^Ge/^68^Ga generator (800 μL) were added. For pH adjustment, 3 M HCl (~25 μL) was used. The reaction mixtures were incubated at 95 °C for 10–15 min. The activity of each preparation was from 750 to 950 MBq, pH 4.5–5. Taking into account the amount of precursor used (5–20 µg, i.e., 4.80–19.19 nmol of PSMA-617 (M = 1042 g/mol) or 3.48–13.94 nmol of Conjugate-1 (M = 1435 g/mol)), this corresponds to a molar activity at the end of synthesis of 39–198 MBq/nmol for [^68^Ga]Ga-PSMA-617 and 54–273 MBq/nmol for [^68^Ga]Ga-Conjugate-1. The resulting solution was cooled, then passed through a sterilizing filter (0.22 μm) and analyzed by radioTLC and radio/UV-HPLC.

For TLC analysis, the ITLC-SG paper (Thermo Fisher Scientific, Waltham, MA, USA) with 0.5 M CH_3_COONH_4_ in a methanol–water mixture (1:1) as solvent was used. Radiography of the TLC strips was performed using a miniGita radio–TLC scanner (Elysia-Raytest, Straubenhardt, Germany).

HPLC analysis of the preparations was carried out using a LicArt-62 chromatograph (Labconcept LLC, Saint Petersburg, Russia), equipped with a diode array detector and flow radioactivity detector GABI Nova basic (2 × 2″ NaI-PMT detector, Elysia-Raytest, Straubenhardt, Germany) with a 5 µL measuring cell. The reversed-phase C_18_ column (Luna^®^, 150 × ⌀4 mm, 5 μm, 100 Å) was purchased from Phenomenex^®^ (Torrance, CA, USA). Gradient flow (0.75 mL min^−1^): 0–5–15–20 min = 17–25–25–17%B, (solvent A was 0.1% (*v*/*v*) TFA in water (TFA—trifluoroacetic acid HPLC grade Sigma-Aldrich/Merck (St. Louis, MO, USA)), and solvent B was 0.1% (*v*/*v*) TFA in acetonitrile (Panreac Quimica (Barcelona, Spain))).

No additional sample purification or reformulation steps (including the use of C18 cartridges) were performed; for *in vitro* and *in vivo* experiments, both radioconjugates were administered immediately following synthesis and quality control (within 25–30 min).

### 4.4. Creation of CT26-PSMA Cell Line and RT-qPCR

The Sleeping Beauty (SB) transposon system was used to breed a stable cell line CT26-PSMA [52,53]. The pSBCXIP plasmid for gene transfer was created in the laboratory based on pQCXIP (Clontech, San Jose, CA, USA) and pSBtet-GP (Addgene, Watertown, MA, USA) vectors. Human prostate adenocarcinoma cells of the LNCaP line served as donors of human PSMA cDNA. The cloned sequence encoded a mature form of the protein corresponding to the annotated sequence NP_004467.1 (https://www.ncbi.nlm.nih.gov/protein (accessed on 16 August 2026)).

Cell line CT26 (BALB/c mouse colon carcinoma) served as the basis for genetically engineered cells. The cell culture was grown on DMEM medium with 4.5 g/L glucose and 25 mM HEPES (PanEco, Moscow, Russia) supplemented with 5% fetal calf serum (BioWest, Nuaillé, France). Cells were cultured in plates and plastic flasks (Jet Biofil, Guangzhou, China) in thermostats at 37 °C, 100% humidity, and 6% CO_2_ in the air phase. Cells were detached from the surface by treatment with trypsin (0.05%) and EDTA (0.02%) in PBS.

CT26 cells were cotransfected with plasmids pSBCXIP carrying the human PSMA gene and puromycin resistance gene and SB100x in pCAG globin pA encoding transposase (Addgene, Watertown, MA, USA) by the calcium–phosphate method. Transfected cells were selected by adding the selective antibiotic puromycin (4 μg/mL) to the growth medium.

For identification of cell populations expressing the human PSMA biomarker, flow cytometry was used. For this, the cells were incubated with phycoerythrin-labeled antibodies (BL 342504, Biolegend, San Diego, CA, USA or IG MA1-10336, Invitrogen, Carlsbad, CA, USA) or with isotype control antibodies (BioLegend, USA) and analyzed on a BD FACSAriaIII flow cytometer using BD FACSDiva software version 7.0 (BD Bioscience, Franklin Lakes, NJ, USA).

Total RNA was isolated from CT26, CT26-PSMA, and LNCaP cells using TRIzol Reagent (Life Technologies, Carlsbad, CA, USA), according to the manufacturer’s recommendations. cDNA was synthesized with random primers and M-MuLV RNase revertase (SibEnzyme, Novosibirsk, Russia). The primers for human FOLH1 (PSMA) forward (ccattagggttaccagacaggc) and reverse (ccctgcatacttgttgtggc) and for GAPDH as the reference gene were obtained from the Harvard Medical School primer bank (Boston, MA, USA). Quantitative PCR was performed using a CFX96 Touch thermal cycler (Bio-Rad, Hercules, CA, USA). Relative expression was calculated by the ΔCt method against GAPDH, with each sample measured in three technical replicates. Measurements were performed 55 days after transfection and repeated after 16 consecutive passages in puromycin-free medium.

### 4.5. Cell Cultures and In Vitro Studies

CT26-PSMA cells were maintained in DMEM (PanEco Ltd., Moscow, Russia) supplemented with 10% fetal bovine serum (Biological Industries, Beit HaEmek, Israel), 2 mM L-glutamine, and puromycin at 4 µg/mL (Gibco, Grand Island, NY, USA). LNCaP cells were maintained in Opti-MEM (Gibco) supplemented with 5% fetal bovine serum and 2 mM L-glutamine, *without* puromycin, since this line carries no resistance cassette. Both lines were grown as monolayers at 37 °C in a humidified atmosphere containing 6% CO_2_. Subconfluent cells were harvested with trypsin (0.05%)/EDTA (0.02%) in PBS.

For saturation binding assays, LNCaP and CT26-PSMA cells were seeded in 12-well plates and grown to 80–90% confluence. Assays were performed at 4 °C to permit binding while suppressing internalization. For each radioconjugate, two parallel series were run. In the total binding series, cells received increasing concentrations (0.1–100 nM) of the ^68^Ga-labeled conjugate in 1 mL of binding buffer and were incubated for 60 min at 4 °C. In the non-specific binding series, cells were pre-incubated with 1 µM 2-PMPA for 30 min at 4 °C and the same radioconjugate concentrations were then added without removing the inhibitor. Because the Ki of 2-PMPA for PSMA is in the sub-nanomolar range, 1 µM raises the apparent K_d_ by more than three orders of magnitude and suppresses specific binding to below 1% even at the highest radioligand concentration. Specific binding was obtained by subtracting non-specific from total binding at each concentration. After incubation, the supernatant was collected, and the cells were washed twice with ice-cold Hanks’ balanced salt solution and trypsinized. The radioactivity of the cell pellets and supernatants was measured using an automatic γ-counter (Wizard 2480, PerkinElmer, Shelton, CT, USA), and saturation binding curves were plotted as bound ligand versus free ligand. Specific binding was plotted against free ligand concentration and K_d_ and B_max_ were obtained by non-linear regression using the one-site-specific binding model in GraphPad Prism (version 11). Receptor density per cell was calculated from B_max_ (pmol per 10^6^ cells) using the Avogadro constant. Each experiment was performed in three technical replicates and repeated in two independent experiments; the confidence intervals reported in Table 1 are those of the non-linear regression fit.

For *in vitro* binding and internalization tests, the cells were seeded into 12-well plates (1 × 10^6^ cells/well, 99% viability) and incubated at 37 °C/6% CO_2_ to form subconfluent monolayers overnight. The day of the experiment, the radiotracer (100 μL of the preparation described in Section 4.3, diluted in complete medium) was added to each well (the final volume of radiotracer with medium is 1 mL per well). Each condition was measured in three technical replicates at each time point (30, 60, and 90 min), and the experiment was repeated in five independent biological replicates (n = 5). After the necessary time of incubation at 37 °C, the supernatant of each well was collected by washing with Hanks’ solution (1 mL) into the marked tubes. Glycine buffer solution (1 mL, 0.2 M Gly, 0.15 M sodium chloride, pH 2.26) was added to the wells for 5 min (4 °C) and then it was collected by washing with Hanks’ solution into the marked tubes. Finally, trypsin solution (1 mL) was added to each well, and then cell lysate was collected by washing with Hanks’ solution into the marked tubes. The radioactivity of all tubes was measured using an automatic γ-counter (Wizard 2480, PerkinElmer). Total cell-associated activity is expressed as a percentage of the added activity per 10^6^ cells; the internalized fraction is expressed as the acid-resistant (lysate) activity as a percentage of total cell-associated activity.

### 4.6. Biodistribution Studies

All experiments involving animals were performed following the ethical standards of Directive 2010/63/EU of the European Parliament and of the Council on the protection of animals used for scientific purposes [54] with permission from the Ethical Board of the State Research Center—Burnasyan Federal Medical Biophysical Center of the Federal Medical Biological Agency.

For the experimental tumor models, athymic male BALB/c Nu mice (7–8 weeks old, 18–20 g; certified laboratory animal nursery of the Institute of Bioorganic Chemistry, Puschino, Russia) were subcutaneously inoculated in the right flank with suspended 4 × 10^6^ CT26-PSMA cells or LNCaP cells in complete medium and 50% Matrigel (Corning Incorporated, Corning, NY, USA). Some tumor samples were transferred for histology to the Pathoanatomic Department of the Burnasyan Federal Medical Biophysical Center (Moscow, Russia).

Mice bearing CT26-PSMA tumors were grouped (groups of each time period of two radiopharmaceuticals, n = 5/group) and injected with 100 μL of each ^68^Ga-PSMA (~5 MBq per mouse) conjugate into the tail vein.

At preselected time points (30, 60, and 90 min) post-injection, animals were sacrificed. The organs of interest were collected and weighed, and the radioactivity was measured using an automatic γ-counter (Wizard 2480, PerkinElmer). All results were decay-corrected using a reference standard of the injected preparation, and the data are expressed as the percentage of injected activity dose per gram of tissue (% IA/g ± SEM) for each organ or tissue. A non-compartmental model was applied to the biodistribution data to derive pharmacokinetic parameters for blood, kidneys, and tumor (effective half-life, min; clearance, μL·s^−1^; area under the curve, ×10^8^ decays·mL^−1^). Detailed pharmacokinetic parameter calculation is described in the Supplementary Materials.

### 4.7. Statistical Analysis

All data are presented as mean ± standard error of the mean (SEM). Unless stated otherwise, n denotes the number of independent biological replicates: n = 5 for the *in vitro* uptake and internalization experiments and for the biodistribution study, with each biological replicate comprising three technical replicates, which were averaged before analysis; n = 10 mice per group for the tumor growth experiment; and, for the saturation binding assays, three technical replicates in each of two independent experiments.

Since the data deviated from normality for several variables and the sample sizes were small, non-parametric methods were used. Comparisons between the two radioconjugates at individual time points were made with the exact Mann–Whitney U-test, and tumor take rates were compared with Fisher’s exact test. Because the three time points comprise independent cohorts of animals rather than repeated measurements, no within-subject factorial analysis was applicable. All analyses were performed in GraphPad Prism (version 11).

The sample size imposes an intrinsic limit on the attainable significance level, which we state explicitly. With n_1_ = n_2_ = 5, there are C(10,5) = 252 possible rank arrangements, so the smallest attainable two-sided exact *p*-value is 2/252 = 0.0079; significance levels below *p* < 0.01 are therefore not attainable in these comparisons and are not reported. A Bonferroni correction across the 11 organs and 3 time points would require *p* < 0.0015, which lies below the smallest attainable value; no family-wise correction was therefore applied, and the reported *p*-values are uncorrected and should be interpreted as descriptive rather than confirmatory. Differences were considered statistically significant at *p* < 0.05.

## 5. Conclusions

The new PSMA-positive transgenic cell line CT26-PSMA was tested and verified positively with [^68^Ga]Ga-labeled conjugates (widely used PSMA-617 and newly synthesized Conjugate-1). CT26-PSMA cells carried the biomarker on a larger proportion of cells than human prostate adenocarcinoma cells of the LNCaP line (95% for CT26-PSMA and 86% for LNCaP) and at a higher membrane density, corresponding to approximately 3.5 × 10^6^ against 0.8 × 10^6^ PSMA molecules per cell. The main value of new transgenic cells is their rapid growth as solid tumors in almost 100% of BALB/c nude mice after inoculation. *In vitro* and *in vivo* studies with [^68^Ga]Ga-labeled conjugates have confirmed the suitability of transgenic CT26-PSMA cells for preclinical investigation of PSMA-directed preparations.

It is important to acknowledge that the CT26-PSMA model, being a genetically engineered murine colon carcinoma cell line, does not recapitulate the full complexity of human prostate cancer biology or its tumor microenvironment. This limitation is shared with other widely used xenograft models, including LNCaP and PC3-PIP. Nevertheless, for the specific purpose of rapid *in vivo* screening and comparative evaluation of PSMA-targeting ligands, the CT26-PSMA model offers a valuable complementary tool to existing prostate cancer cell lines. Moreover, unlike PC3-PIP, which is not commercially available and is distributed primarily through material transfer agreements between research groups, the CT26-PSMA line is offered to academic investigators on request and free of charge, and—being of BALB/c origin—can additionally be engrafted in immunocompetent or minimally immunosuppressed hosts, thereby lowering the barrier for laboratories seeking to establish PSMA-targeted screening platforms.

## Figures and Tables

**Table 1:** Saturation binding data (best-fit values with 95% CI).

Cell Line	Radioconjugate	K_d_, nM	B_max_, pmol/10^6^ Cells	Receptor Density, Sites per Cell
CT26-PSMA	[^68^Ga]Ga-Conjugate-1	11.6 (7.2 to 18.3)	5.8 (5.0 to 6.7)	3.5 × 10^6^
	[^68^Ga]Ga-PSMA-617	10.1 (5.1 to 19.3)	5.7 (4.6 to 7.0)	3.4 × 10^6^
LNCaP	[^68^Ga]Ga-Conjugate-1	9.1 (6.6 to 15.4)	1.4 (1.2 to 1.7)	0.8 × 10^6^
	[^68^Ga]Ga-PSMA-617	9.0 (4.4 to 17.3)	1.3 (1.1 to 1.6)	0.8 × 10^6^

Values are best-fit estimates with the 95% confidence intervals of the non-linear regression (one-site-specific binding model). Each experiment was performed in three technical replicates and repeated in two independent experiments. Receptor density was calculated from B_max_ using the Avogadro constant.

**Table 2:** Calculated values of pharmacokinetic parameters of [^68^Ga]Ga-labeled conjugates (mean ± SEM, n = 5). ** *p* < 0.01 for the difference between [^68^Ga]Ga-PSMA-617 and [^68^Ga]Ga-Conjugate-1 (exact Mann–Whitney U-test).

Pharmacokinetics Parameters	Organ/Tissue	[^68^Ga]Ga-PSMA-617	[^68^Ga]Ga-Conjugate-1
Effective half-time, min	Blood	18.7 ± 0.5	20.3 ± 0.7
	Kidneys	12.9 ± 1.3	27.5 ± 1.3 **
	Tumor	=T_phys_ (67.71)	=T_phys_ (67.71)
Clearance, μL∙s^−1^	Blood	17.6 ± 0.7	23.4 ± 0.6 **
	Kidneys	0.5 ± 0.1	1.6 ± 0.0 **
	Tumor	1.7 ± 0.1	3.5 ± 0.3 **
Area under curve, ×10^8^ decays∙mL^−1^	Blood	2.9 ± 0.1	2.1 ± 0.1 **
	Kidneys	108.9 ± 12.4	31.0 ± 0.8 **
	Tumor	30.0 ± 2.2	14.8 ± 1.3 **

T_eff_ = T_phys_·T_biol_/(T_phys_ + T_biol_), with T_phys_ = 67.71 min for ^68^Ga. Tumor activity concentration was still rising at the final sampling point, so the elimination phase was not observed and T_biol_ could not be estimated; T_biol_ → ∞ was therefore assumed for the tumor, giving T_eff_ = T_phys_. Clearance was calculated as CL = IA/AUC with an injected activity (IA) of ~5 MBq per animal. Tissue density was assumed to be 1 g/mL for tumor and kidney.

**Table 3:** Comparative characterization of various parameters of the most popular cell cultures used in studies of radiolabeled PSMA conjugates.

Cell Culture	PSMA Receptors	Androgen Dependence	Doubling Time	Time to Target Volume	Take Rate	Immunocompetent Host	Availability	Refs
LNCaP	Yes	Dependent	60–72 h	300 mm^3^ (8 weeks)	Mean (10%, nude BALB/c)	No	ATCC	[32,33,34,35], this work
PC3-PIP	Yes (transfected)	Independent	48 h	100–200 mm^3^ (1–2 weeks)	High	No	On request	[29,36,37]
22Rv1	Low PSMA-expressed	Independent	35–40 h	180 mm^3^ (2 weeks)	High (92% orthotopic; 100% intratibial)	No	ATCC	[22,38,39,40]
VCaP	Yes	Sensitive (AR-amplified; responds to castration)	53 h	300 mm^3^ (7 weeks)	Mean (85%, SCID)	No	ATCC	[41,42,43,44]
C4-2	Low PSMA-expressed	Independent	48 h	200 mm^3^ (3–4 weeks, C4-2B)	High (100% intratibial, SCID)	No	ViroMed Laboratories at Johns Hopkins, USA	[22,45,46,47]
CT26-PSMA	Yes (transfected)	Independent	24 h	300 mm^3^ (8–10 days)	Very high (100%)	Yes (BALB/c background)	Available to academic investigators on request, free of charge	[48], this work

## Data Availability

Data will be made available upon reasonable request to the corresponding authors.

## Supplementary Materials

The following supporting information can be downloaded at: https://www.mdpi.com/article/10.3390/ijms27167426/s1.
