# Supplementary material for: Development and Characterization of the PSMA-Expressing CT26-PSMA Cell Line as a Rapid Preclinical Platform for 68Ga-Labeled PSMA-Targeted Radioconjugates
Source: Int J Mol Sci. 2026 Aug 19;27(16):7426. doi: 10.3390/ijms27167426 (PMC13513800; doi:10.3390/ijms27167426)
Supplement: Supplementary file 1 [file ijms-27-07426-s001.zip › ijms-4439811-supplementary.pdf]

Detailed pharmacokinetic parameters calculation

The effective half-lives (T_{eff}) and biological half-lives (T_{biol}) for critical organs and the whole body were calculated using Equation (S1):

$$T_{eff} = \frac{T_{phys} \times T_{biol}}{T_{phys} + T_{biol}} \quad \text{(S1)}$$

where T_{phys} is the physical half-life of the radionuclide (T_{phys} = 67.71 min for ^{68}Ga), and T_{biol} is the biological half-life of the agent.

All activity concentrations used in the fitting were decay-corrected to the time of injection, so that the fitted parameter is the biological half-life; the effective half-life was then obtained from Equation (S1). For the tumor, activity concentration was still rising at the final sampling point (90 min), so the elimination phase was not observed and T_{biol} could not be estimated. We therefore adopted the most conservative assumption available, $T_{biol} \to \infty$, under which Equation (S1) reduces to $T_{eff} = T_{phys}$ = 67.71 min.

The total area under the curve (AUC), which represents the cumulative number of nuclear disintegrations per unit volume of organ or tissue, was calculated by summing the integrated portions (uptake and clearance) of the time–activity curve, as described in Equation (S2):

$$AUC = \int_0^{t_{max}} F_1(t)dt + \int_{t_{max}}^{\infty} F_2(t)dt \quad \text{(S2)}$$

where $F_1(t)$ is the function describing the rapid accumulation phase of the agent in the organ, limited to the time interval [0; t_{max}], and $F_2(t)$ is the function describing the clearance phase from the compartment with a rate constant λ_i.

The clearance (CL), reflecting the elimination of the radiopharmaceutical through excretion or biotransformation, was calculated using Equation (S3):

$$CL = \frac{IA}{AUC} \quad \text{(S3)}$$

where IA is the injected activity (MBq or kBq), taken as ~5 MBq per animal in the present study. Tissue density was assumed to be 1 g/mL for tumour and kidney.

Model-independence check and limitations of the analysis. Because the first sampling point was at 30 min, the accumulation phase $F_1(t)$ is model-based, and the renal AUC in particular is sensitive to the assumed shape of the uptake function before that point; the AUC values should therefore be regarded as comparative rather than absolute measures of exposure. To verify that the comparison between the two radioconjugates does not depend on this assumption, the measured activity concentrations were also integrated directly over the observation window by the trapezoidal rule, with decay correction and the conservative assumption of zero uptake at t = 0. The resulting [^{68}Ga]Ga-PSMA-617 / [^{68}Ga]Ga-Conjugate-1 AUC ratios were 1.31 for blood, 2.25 for the kidneys and 1.87 for the tumour, in agreement in direction and order of magnitude with the model-derived ratios of 1.38, 3.51 and 2.03 respectively.

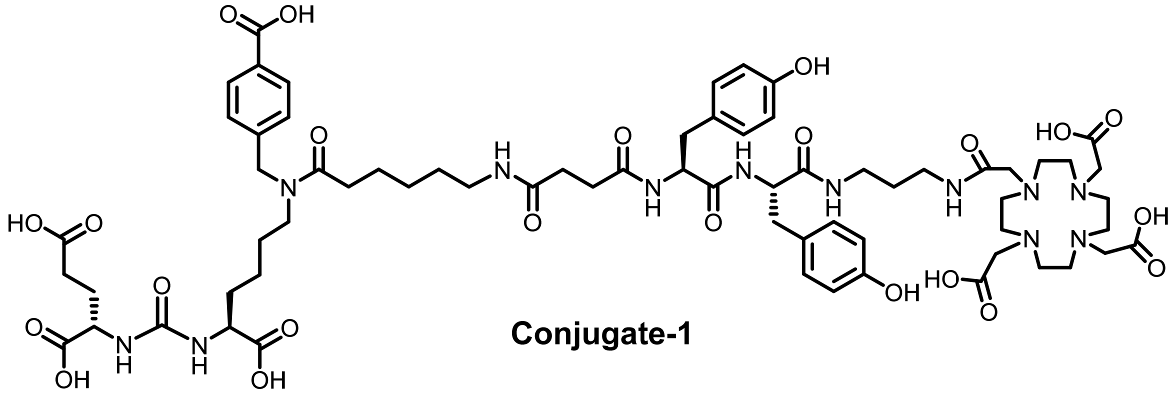

Figure S1. Chemical structure of Conjugate-1.

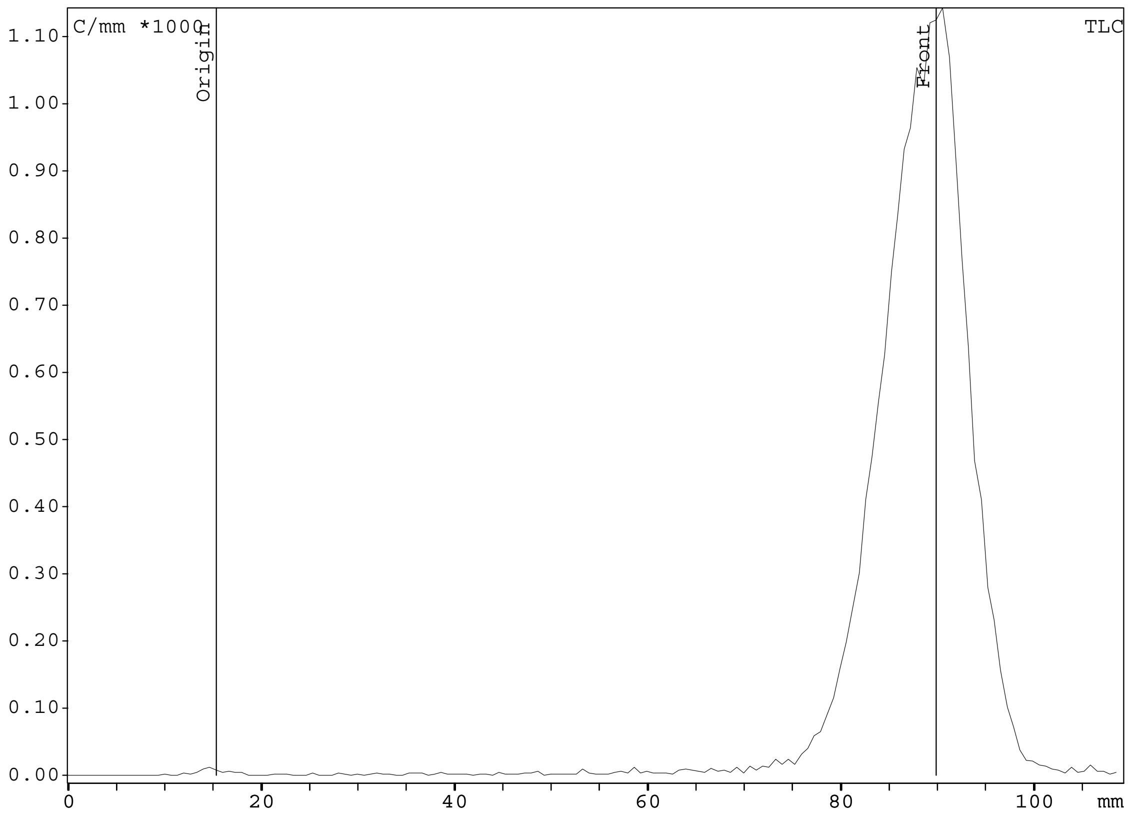

Figure S2. The typical radio-TLC chromatogram of [^{68}Ga]Ga-PSMA-617 preparation.

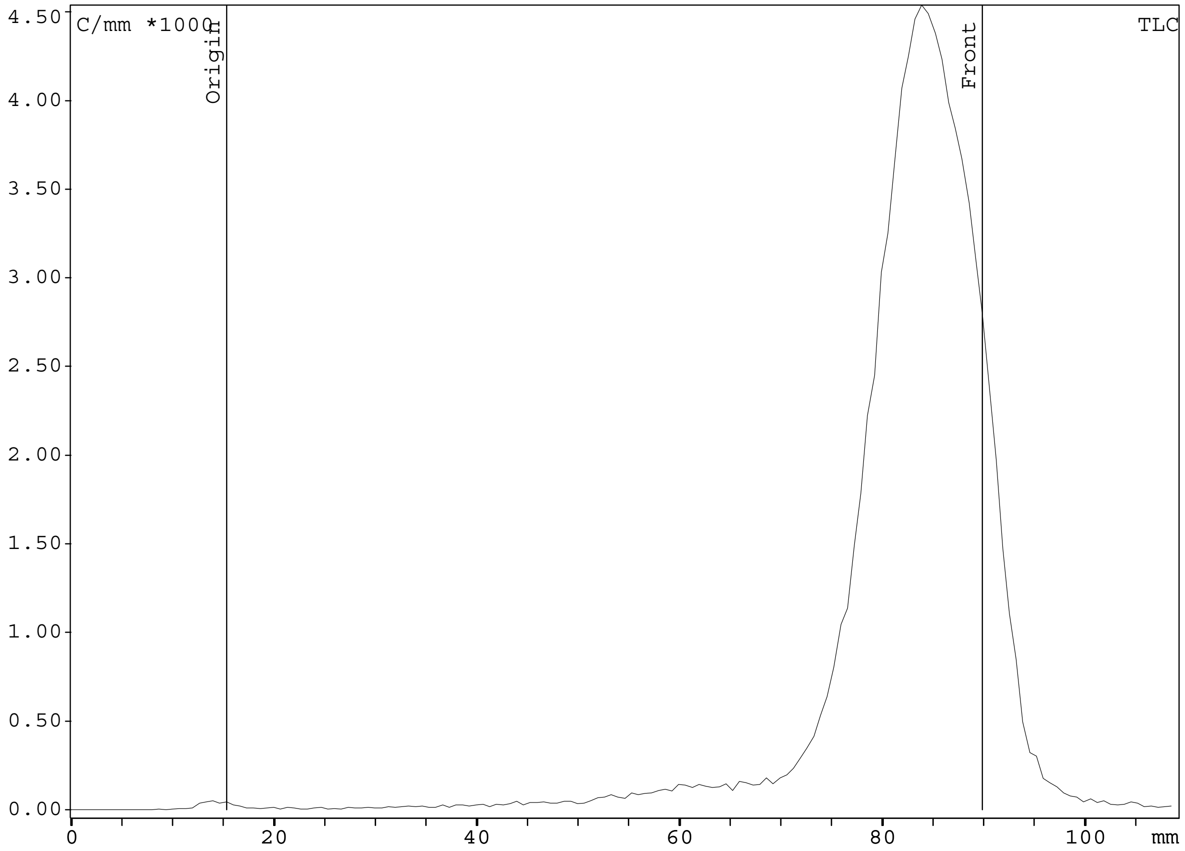

Figure S3. The typical radio-TLC chromatogram of [^{68}Ga]Ga-Conjugate-1 preparation.

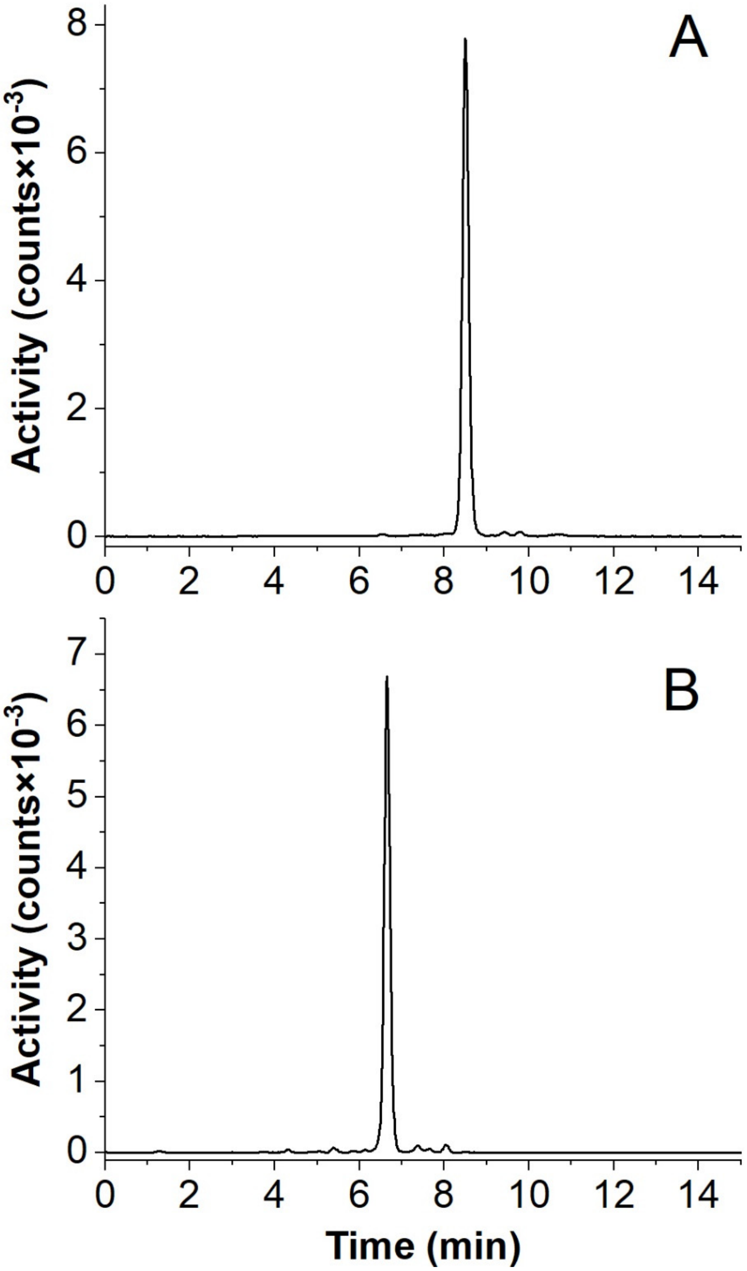

Figure S4. The typical radio-HPLC chromatograms of [^{68}Ga]Ga-PSMA-617 (A) and [^{68}Ga]Ga-Conjugate-1 (B).

Table S1. *In vitro* uptake of [^{68}Ga]Ga-labeled conjugates in CT26-PSMA cell culture (mean ± SEM, n = 5). * $p < 0.05$ and ** $p < 0.01$ for the difference between [^{68}Ga]Ga-PSMA-617 and [^{68}Ga]Ga-Conjugate-1 (exact Mann–Whitney U-test).

Time after activity adding, min	PSMA-617, % of added activity	PSMA-617, Internalized %	Conjugate-1, % of added activity	Conjugate-1, Internalized %
30	3.76 ± 0.22	6.12 ± 1.05	4.22 ± 0.12 *	9.13 ± 0.94

60	2.96 ± 0.33	9.12 ± 1.60	4.78 ± 0.30 **	11.92 ± 0.31 *
90	3.05 ± 0.22	7.67 ± 1.33	4.84 ± 0.27 **	7.69 ± 0.92

"% of added activity" is the total cell-associated activity per 10^6 cells, expressed as a percentage of the activity added to the well. "Internalized %" is the acid-resistant (lysate) fraction expressed as a percentage of the total cell-associated activity. With n = 5 per group the smallest attainable two-sided p-value under the exact Mann–Whitney U-test is 0.0079, so $p < 0.01$ is the highest attainable significance level; the p-values are uncorrected for multiplicity.

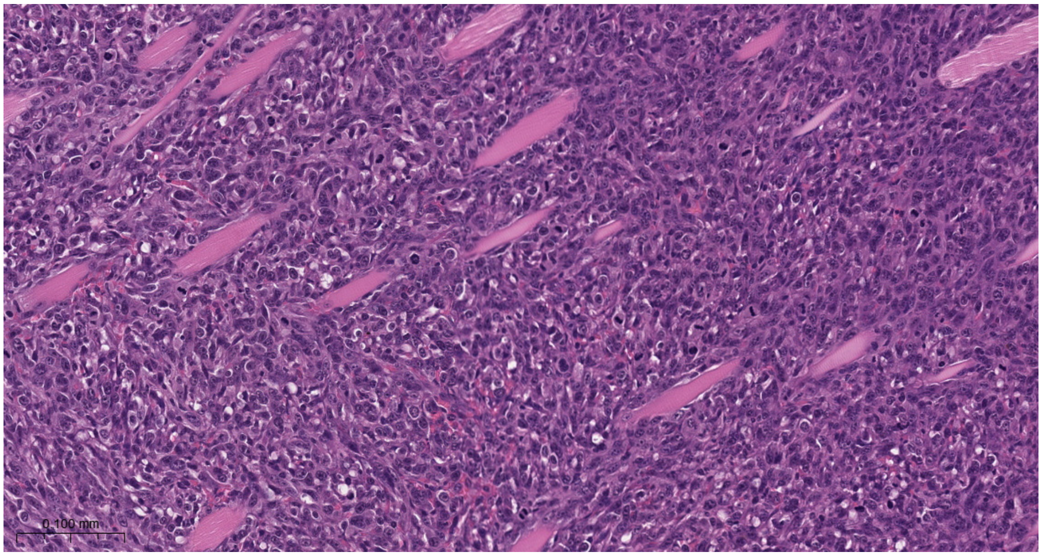

Figure S5. Histology of CT26-PSMA tumor

Adipose and striated muscle tissue fragments exhibit invasive malignant tumor growth characterized by dense, solid layers of atypical epithelioid cells with intensely eosinophilic cytoplasm, suggesting aggressive behavior. These atypical cells display significant nuclear polymorphism, with nuclei that vary widely in size and shape. Additionally, there is a high mitotic index, indicating a large number of mitotic figures, which points to a rapid rate of cell division and tumor proliferation. The observed histological features, including marked cellular atypia, nuclear polymorphism, and frequent mitoses, align with a diagnosis of poorly differentiated carcinoma. This carcinoma type generally lacks clear structural differentiation and often signifies a more advanced and aggressive tumor, potentially associated with a poor prognosis and high metastatic potential.

Table S2. *In vivo* uptake of [^{68}Ga]Ga-labeled conjugates in athymic mice (mean ± SEM, n = 5). * $p < 0.05$ and ** $p < 0.01$ for the difference between [^{68}Ga]Ga-PSMA-617 and [^{68}Ga]Ga-Conjugate-1 (exact Mann–Whitney U-test).

**Organs / tissues**	**Gallium-68-labeled conjugates uptake, % of injected activity per gram**					
	**PSMA-617**			**Conjugate-1**		
	**30 min**	**60 min**	**90 min**	**30 min**	**60 min**	**90 min**
Blood	1.73 ± 0.22	0.59 ± 0.09	0.34 ± 0.01	1.25 ± 0.20	0.53 ± 0.06	0.30 ± 0.06
Lungs	1.69 ± 0.17	0.50 ± 0.10	0.24 ± 0.05	0.66 ± 0.20	0.46 ± 0.07	0.25 ± 0.05
Heart	0.83 ± 0.17	0.27 ± 0.05	0.13 ± 0.03	0.64 ± 0.07	0.33 ± 0.13	0.19 ± 0.07
Salivary glands	0.50 ± 0.13	0.62 ± 0.48	0.43 ± 0.14	0.65 ± 0.38	0.27 ± 0.05	0.21 ± 0.09

Stomach	0.74 ± 0.26	0.26 ± 0.04	0.12 ± 0.05	0.61 ± 0.05	0.16 ± 0.05	0.10 ± 0.02
Liver	0.42 ± 0.03	0.16 ± 0.03	0.10 ± 0.02	0.29 ± 0.08	0.16 ± 0.03	0.13 ± 0.03
Kidneys	50.47 ± 3.06	12.89 ± 5.22	3.86 ± 2.42	18.41 ± 2.55 **	8.83 ± 1.28 *	7.65 ± 2.10 **
Spleen	0.50 ± 0.10	0.28 ± 0.13	0.15 ± 0.06	0.81 ± 0.30	0.24 ± 0.18	0.15 ± 0.05
Prostate	0.57 ± 0.35	0.43 ± 0.07	0.27 ± 0.07	0.44 ± 0.18	0.40 ± 0.17	0.20 ± 0.08
Muscle	0.32 ± 0.09	0.44 ± 0.19	0.18 ± 0.03	0.35 ± 0.05	0.13 ± 0.12	0.08 ± 0.06
CT26-PSMA tumor	10.79 ± 1.97	14.25 ± 7.04	13.08 ± 3.28	5.51 ± 0.33 **	7.50 ± 1.60	8.13 ± 3.72 **
